# Supplementary material for: Functional Block of Interleukin-6 Reduces a Bone Pain Marker But Not Bone Loss in Hindlimb-Unloaded Mice
Source: Int J Mol Sci. 2020 May 15;21(10):3521. doi: 10.3390/ijms21103521 (PMC7278999; doi:10.3390/ijms21103521)
Supplement: Supplementary file 1 [file ijms-21-03521-s001.pdf]

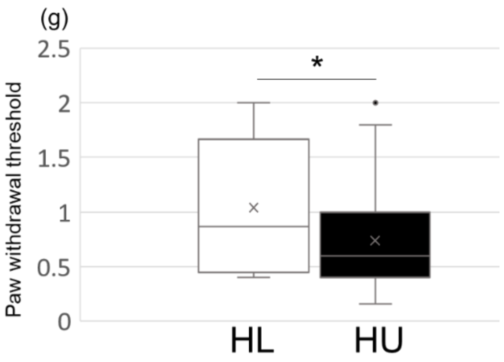

Figure S1. Paw withdrawal threshold after a 2-week tail suspension.

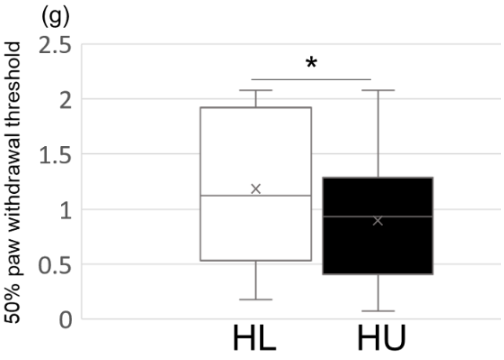

Figure S2. 50% paw withdrawal threshold after a 2-week tail suspension.

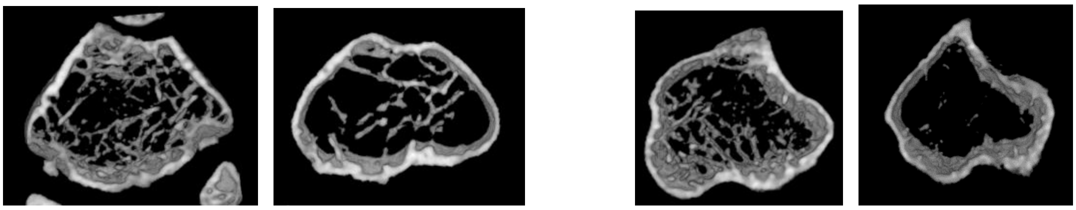

Figure S3. Three-dimensional images of distal femoral and proximal tibial metaphysis after a 2-week tail suspension.

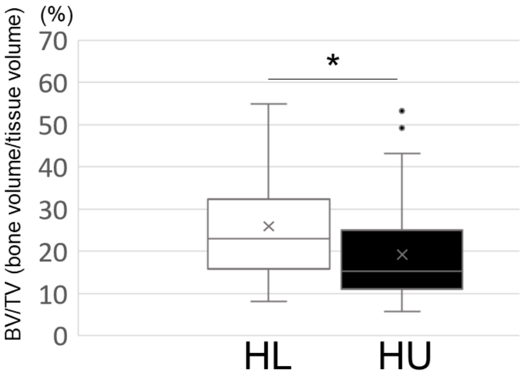

Figure S4. BV/TV of distal femoral metaphysis after a 2-week tail suspension.

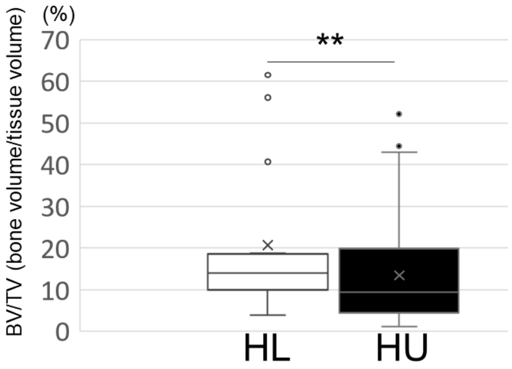

Figure S5. BV/TV of proximal tibial metaphysis after a 2-week tail suspension.
